# Characterization of Circulating MicroRNA Expression in Patients with a Ventricular Septal Defect

**DOI:** 10.1371/journal.pone.0106318

**Published:** 2014-08-28

**Authors:** Dong Li, Long Ji, Lianbo Liu, Yizhi Liu, Haifeng Hou, Kunkun Yu, Qiang Sun, Zhongtang Zhao

**Affiliations:** 1 Department of Epidemiology and Health Statistics, School of Public Health, Shandong University, Jinan, Shandong Province, China; 2 School of Public Health, Taishan Medical University, Tai’an, Shandong Province, China; 3 Tai’an Children’s Hospital, Tai’an, Shandong Province, China; Queen’s University Belfast, United Kingdom

## Abstract

**Objectives:**

Ventricular septal defect (VSD), one of the most common types of congenital heart disease (CHD), results from a combination of environmental and genetic factors. Recent studies demonstrated that microRNAs (miRNAs) are involved in development of CHD. This study was to characterize the expression of miRNAs that might be involved in the development or reflect the consequences of VSD.

**Methods:**

MiRNA microarray analysis and reverse transcription-polymerase chain reaction (RT-PCR) were employed to determine the miRNA expression profile from 3 patients with VSD and 3 VSD-free controls. 3 target gene databases were employed to predict the target genes of differentially expressed miRNAs. miRNAs that were generally consensus across the three databases were selected and then independently validated using real time PCR in plasma samples from 20 VSD patients and 15 VSD-free controls. Target genes of validated 8 miRNAs were predicted using bioinformatic methods.

**Results:**

36 differentially expressed miRNAs were found in the patients with VSD and the VSD-free controls. Compared with VSD-free controls, expression of 15 miRNAs were up-regulated and 21 miRNAs were downregulated in the VSD group. 15 miRNAs were selected based on database analysis results and expression levels of 8 miRNAs were validated. The results of the real time PCR were consistent with those of the microarray analysis. Gene ontology analysis indicated that the top target genes were mainly related to cardiac right ventricle morphogenesis. NOTCH1, HAND1, ZFPM2, and GATA3 were predicted as targets of hsa-let-7e-5p, hsa-miR-222-3p and hsa-miR-433.

**Conclusion:**

We report for the first time the circulating miRNA profile for patients with VSD and showed that 7 miRNAs were downregulated and 1 upregulated when matched to VSD-free controls. Analysis revealed target genes involved in cardiac development were probably regulated by these miRNAs.

## Introduction

Congenital heart disease (CHD) is cardiovascular malformation caused by abnormal development of the fetal heart and the great vessels. Its incidence is 32.74/10000 in China [1] but 5.4-16.1/1000 in the other countries [2], [3]. CHD is one of the most common types of congenital malformation in children, and is also one of the main causes of neonatal and infant mortality [4].

The causes of CHD are complex, and are not fully understood. Most CHD (90%) may be related to genetic and environmental factors [5]. The common CHDs include atrial septal defect (ASD), ventricular septal defect (VSD), patent ductus arteriosus (PDA), tetralogy of Fallot (TOF), transposition of the great arteries (TGA), pulmonary valve atresia (PA), coarctation of the aorta (COA) and tricuspid atresia (TA). Among them, VSD is one of the most common, accounting for about 20% of CHD [6]. VSD is mainly caused by left and right ventricular septal defect-induced abnormal traffic. Despite the fact that its embryology and physiology have been elucidated, its etiology and pathogenesis are unclear [7].

A microRNA (miRNA) is a post-transcriptional regulatory factor and small single-stranded non-coding RNA molecule, 18–22 nucleotides in length. It can pair with 3′ non-coding regions of a target gene’s mRNA, and negatively regulate expression of target genes at the posttranscriptional level. It can regulate cell growth, metabolism, differentiation and apoptosis, participating in the growth of the living organism [8]. miRNA plays an important role in many physiological and pathological processes. miRNA genes represent only 1% of human genes [9]. However, sequence analysis suggests that miRNA can potentially regulate 30% of human genes through complex regulatory networks [10]. The miRNA stably expressed in body fluids plays an important role in cardiovascular diseases and tumor occurrence, and circulating miRNA can be used as a potential biomarker for disease diagnosis as it is very stable in serum and cannot be degraded by RNA degrading enzymes [11]–[14]. Recent studies showed that miRNA is involved in embryonic heart development, morphogenesis of the heart, and myocardial cell growth and differentiation, playing an important function in the occurrence and development of cardiovascular disease [15]. It was reported that miRNA is associated with the pathogenesis of CHD [16], and it plays an increasingly important role in diagnosis and treatment of heart-related diseases [17], [18]. MiR-1 and miR-133 can control development of the myocardium and the skeleton [19]. Huang et al. [20] found that miRNAs may play a central role in craniofacial and cardiovascular systems, and if mutated may cause nerve - craniofacial - congenital heart defects. A number of target genes regulated by miRNA that might be related with VSD have been identified. For example, miRNA -21 and miRNA-181a play an important role in the occurrence and development of VSD in mice with VSD phenotype after Dicer gene knockout [20]. A very recent study has also found that two miRNAs, miR-1-1 and miR-181c, are associated with VSD pathogenesis [21]. However, although there are many miRNAs related to the occurrence and development of VSD, it is unclear how they regulate VSD, and more research is needed.

miRNA microarray analysis for gene expression profiles is one of the most important methods used to screen and study the occurrence and development of disease-associated specific miRNAs. We used it here to screen differentially expressed miRNAs in the plasma of patients with VSD and that of VSD-free controls. Meanwhile, quantitative real-time fluorescent polymerase chain reaction (RT-PCR) was used to verify the reliability of miRNA microarray analysis in detecting differentially expressed miRNAs. In addition, we predicted downstream target genes regulated by differentially expressed miRNAs using target gene prediction software, and further analyzed its biological function. It is hoped that this study will provide valuable information for clarifying the pathogenesis of VSD at the molecular level.

## Patients and Methods

### Participant selection

The study was approved by the Ethics Committee of Taishan Medical College and performed with the written informed consent of all the patients and guardians.

Patients with VSD and VSD-free participants were selected from the Children’s Hospital of Jinan City (Shandong, China) between August 2012 and June 2013. Patients with VSD were enrolled from surgical cardiovascular patients with CHD (shunt diameter of 5–8 mm) diagnosed by ultrasound technique or by intraoperative observation. All the patients had VSD but without other deformities. Meanwhile, the VSD-free participants in the control group were selected from hospitalized orthopedic patients. They were from the same areas as the patients with VSD, but had no CHD genetic history. One patient suffered from a fracture of the clavicle while the other two suffered from subluxation of the radial head.

Three patients with VSD or VSD-free participants were selected for miRNAs microarray analysis. 20 patients with VSD and 15 VSD-free participants were selected to verify the differentially expressed miRNAs using RT-PCR.

### Blood sample collection and plasma preparation

The fasting blood sampling was performed in the morning. Venous blood (5 mL) was collected from each participant in an ethylenediaminetetraacetic acid (EDTA) tube, allowed to stand for 10 min, and then centrifuged at 1500 rpm for 10 min. The upper light yellow liquid was harvested, which was plasma. This was then aliquoted into RNase-free epoxy resin tubes and kept at −80°C.

### Plasma RNA extraction and quality detection

The total RNA in 250 µl plasma was extracted following instructions of the TRIzol and miRNeasy mini kit (Qiagen, USA). The RNA was detected by Thermo Scientific NanoDrop 1000 Spectrophotometer and its purity was assessed by the ratio of absorbance (OD value  =  A260/A280) at 260 and 280 nm. A value between 1.8 and 2.1 was considered as acceptable.

### Plasma miRNA labeling and isolation

The miRCURY Hy3/Hy5 Power labeling kit (Exiqon, Vedbaek, Denmark) was used for miRNA labeling. Each sample was 3′-end-labeled with the Hy3 fluorescent label using RNA ligase following the instructions of the labeling kit to obtain fluorescent probes for microarray hybridization.

The miRNA was isolated according to the kit instructions. Specifically, 2 µl RNA was dissolved in water, mixed with 1 µl calf intestinal phosphatase (CIP) buffer and CIP. The mixture was incubated at 37°C for 30 min, and terminated by incubation at 95°C for 5 min. Then, 3 µl labeling buffer, and l.5 µl fluorescent label (Hy3), 2 µl DMSO, and 2 µl labeling enzyme were added to the mixture. The labeling reaction was incubated at 16°C for 1 h and then terminated at 65°C for 15 min.

### Plasma miRNA microarray hybridization

The Hy3 labeled sample was hybridized on a miRCURYTM LNA Array (v.18.0) (Exiqon, Denmark) according to array manual. The total 25 µl mixture from Hy3 labeled sample was mixed with 25 µl hybridization buffer, denatured at 95°C for 2 min, incubated on ice for 2 min, and then hybridized to the miRCURYTM microarray for 16–20 hours at 56°C in a phalan hybridization bag.

### Microarray scanning and image data processing

A GenePix 4000B microarray scanner (Axon Instruments, Foster City, CA) was used to scan the microarray, and collect fluorescence intensity. Genepix Pro 6.0 software (Axon) was used for image analysis. The original signal of each probe was corrected by subtracting that of the background. Probe intensities were corrected by subtracting background intensity and normalized using median array normalization, where each background-subtracted intensity was divided by the median intensity of non-control probes with corrected value≥50 on the array. Then, the fold change in miRNA expression between VSD group and control group was calculated for each probe (VSD group/control group). The fold change of >1.5 was used to screen differentially expressed miRNA. All microarray data reported in the manuscript have been deposited at GEO (accession number: GSE54675).

### Plasma miRNA target gene prediction

Three databases including targetscan (http://www.microrna.org/microrna/home.do), mirbase (http://pictar.mdc-berlin.de), and Miranda (http://www.targetscan.org/vert_60/) were used to predict target genes for the miRNAs. 36 differentially expressed miRNAs were identified by microarray analysis. 15 miRNAs corresponding to 447 target genes were obtained from three databases including targetscan, mirbase, and Miranda. These 15 miRNAs were selected for further study.

### Correlation between miRNAs and target genes

The 447 target genes regulated by 15 miRNA were annotated using the Gene Ontology (GO) database (http://www.geneontology.org/) to obtain all functional annotations of target genes. Fisher’s exact test was then used to calculate significance level (*P*-value) of each function, which was used to screen genes with significant functions. A function with a *P*-value≤0.05 was considered as significant. The smaller the *P* value was the more significant the function of GO was.

### Verifying differentially expressed miRNAs with RT-PCR

The hsa-miR-93 miRNA was used as an internal reference, to which the results of other microRNAs were normalized because it was stable and expressed similarly between samples. Primer sequences for all microRNAs were designed based on microRNA sequences from the miRBase database. 100 ng of total RNA was used in 20 µL of the reverse-transcription reaction containing 2 µL of 10×reverse-transcription buffer, 0.3 µL of RNase inhibitor (40 U/µL), 2 µL dNTP(2.5 mM each), 0.2 µL of multiscribe reverse-transcriptase (200 U/µl, EPICENTRE Biotechnologies, USA), 0.3 µL of miRNA-specific primers (1 Um) and adding H_2_O upto 20 µL. RT was carried out using a Gene Amp PCR System 9700 (Applied Biosystems, USA) at 16°C for 30 min followed by 42°C for 40 min and then inactivated at 85°C for 5 min. After this the reaction was placed on ice ready for use. Meanwhile, 2 µl of RT products was added into an 8 µl real-time PCR assay reagents containing 5 µL of 2× PCR Master Mix(Superarray, USA), 1 µL of primers and 2 µL of water, Real-time PCR was carried out on an Applied BioSystems PRISM7900 thermocycler at 95°C denaturation for 10 min, 95°C for 10 s, 60°C for 60 s, and 95°C for 15 s. Fluorescence intensity was detected. The reaction was repeated for 40 cycles, then slowly heated from 60°C to 99°C. The melting curve was plotted and data was analyzed with 2^−△△CT^ method. The RT primers and the PCR primer sets for each microRNA amplification are shown in Table 1.

**Table 1:** Sequences of the primers used in the reverse transcription and RT-PCR.

Primer name	Primer sequence
hsa-miR-155-5p RT	5′GTCGTATCCAGTGCGTGTCGTGGAGTCGGCAATTGCACTGGATACGACACCCCTA3′
hsa-miR-155-5p: F	5′GGGGTAATGCTAATCGTGA3′
hsa-miR-155-5p: R	5′CAGTGCGTGTCGTGGAG3′
hsa-miR-222-3p RT	5′GTCGTATCCAGTGCGTGTCGTGGAGTCGGCAATTGCACTGGATACGACACCCAGT3′
hsa-miR-222-3p: F	5′GGGAGCTACATCTGGCTA3′
hsa-miR-222-3p: R	5′TGCGTGTCGTGGAGTC3′
rno-miR-379-5p RT	5′ GTCGTATCCAGTGCGTGTCGTGGAGTCGGCAATTGCACTGGATACGACCCTACG3′
rno-miR-379-5p: F	5′GGGGTGGTAGACTATGGAA3′
rno-miR-379-5p: R	5′CAGTGCGTGTCGTGGAGT3′
hsa-miR-409-3p RT	5′ GTCGTATCCAGTGCGTGTCGTGGAGTCGGCAATTGCACTGGATACGACAGGGGT3′
hsa-miR-409-3p: F	5′GGAATGTTGCTCGGTGA3′
hsa-miR-409-3p: R	5′CAGTGCGTGTCGTGGA3′
hsa-miR-433 RT	5′GTCGTATCCAGTGCGTGTCGTGGAGTCGGCAATTGCACTGGATACGACACACCG3′
hsa-miR-433: F	5′CAATATCATGATGGGCTCCT3′
hsa-miR-433: R	5′CAGTGCGTGTCGTGGAGT3′
hsa-miR-498 RT	5′GTCGTATCCAGTGCGTGTCGTGGAGTCGGCAATTGCACTGGATACGACGAAAAAC3′
hsa-miR-498: F	5′GGTTTCAAGCCAGGGG3′
hsa-miR-498: R	5′CAGTGCGTGTCGTGGAG3′
hsa-let-7e-5p RT	5′GTCGTATCCAGTGCGTGTCGTGGAGTCGGCAATTGCACTGGATACGACAACTAT3′
hsa-let-7e-5p: F	5′GGGGTGAGGTAGGAGGTTGT3′
hsa-let-7e-5p: R	5′GTGCGTGTCGTGGAGTCG3′
hsa-miR-487b RT	5′GTCGTATCCAGTGCGTGTCGTGGAGTCGGCAATTGCACTGGATACGACAAGTGG3′
hsa-miR-487b: F	5′GGGGAATCGTACAGGGTCAT3′
hsa-miR-487b: R	5′GTGCGTGTCGTGGAGTCG3′

**RT:** reverse-transcription; F: forward primer; R: reverse primer.

### Statistical analysis

All the data were expressed as mean ± standard deviation, and analyzed using SPSS 16.0 software (SPSS Inc, USA). The *t* test was used for intergroup data comparison. For the microarray data, after normalization, the statistical significance of differentially expressed miRNA was analyzed by *t* test. For all of the analyses, a probability smaller than 0.05 (*P*<0.05) was considered statistically significant. *P* values were corrected for multiple testing by false discovery rate (FDR).

## Results

The background characteristics of the VSD patients and VSD-free controls used in the miRNA microarray are shown in Table 2. The three VSD patients were matched in terms of gender, age, place of birth, method of birth, history of taking contraceptives, and history of CHD with those of VSD-free controls. The background characteristics of the 20 patients with VSD and the 15 age- and gender-matched VSD-free controls are summarized in Table 3. The participants in the two groups were significantly different in the history of abortion, but not in the other variables (*P*>0.05).

**Table 2:** Background characteristics of the three patients with VSD and the three matched VSD-free controls analyzed with the miRNA microarray study.

	VSDpatientD28	MatchedControlC58	VSDpatientD55	MatchedcontrolC56	VSDPatientD61	MatchedControlC82
Gender	male	male	male	male	female	female
Age (years)	1.2	1.2	2.1	2.1	1.4	1.4
Place of birth	Jinan,Shandong,China	Jinan,Shandong,China	Dezhou,Shandong,China	Dezhou,Shandong,China	Taian,Shandong,China	Taian,Shandong,China
Method of birth	Natural birth	Natural birth	Natural birth	Natural birth	Natural birth	Natural birth
History oftakingcontraceptive	No	No	Yes	Yes	No	No
History oftaking Folicacid	Yes	Yes	Yes	Yes	No	No
History of CHD	No	No	No	No	No	No

VSD, ventricular septal defect; CHD, congenital heart disease; miRNA, microRNA.

**Table 3:** Clinical data for the 20 patients with VSD and the 15 VSD-free controls.

Variables	VSD (n = 20)	Control (n = 15)	*P*-Value
Male/female	11/9	9/6	0.573
Age (years)	1.23±0.51	1.28±0.37	0.354
Gestational age (weeks)	38.35±2.76	39.27±0.88	0.121
Birth weight (kg)	3.19±0.50	3.12±0.50	0.804
History of abortion, n (%)	9(45.00)	1(6.67)	<0.001
History of taking contraceptive, n (%)	2(10.00)	1(6.67)	0.497
History of CHD, n (%)	0(0%)	0(0%)	>0.05
Maternal age (years)	25.3±4.86	27.13±4.61	0.58
History of taking Folic acid, n (%)	17(85.00)	14(93.33)	0.125

VSD, ventricular septal defect; CHD, congenital heart disease.

### Plasma miRNA expression determined by miRNA microarray analysis

**Figure 1:**
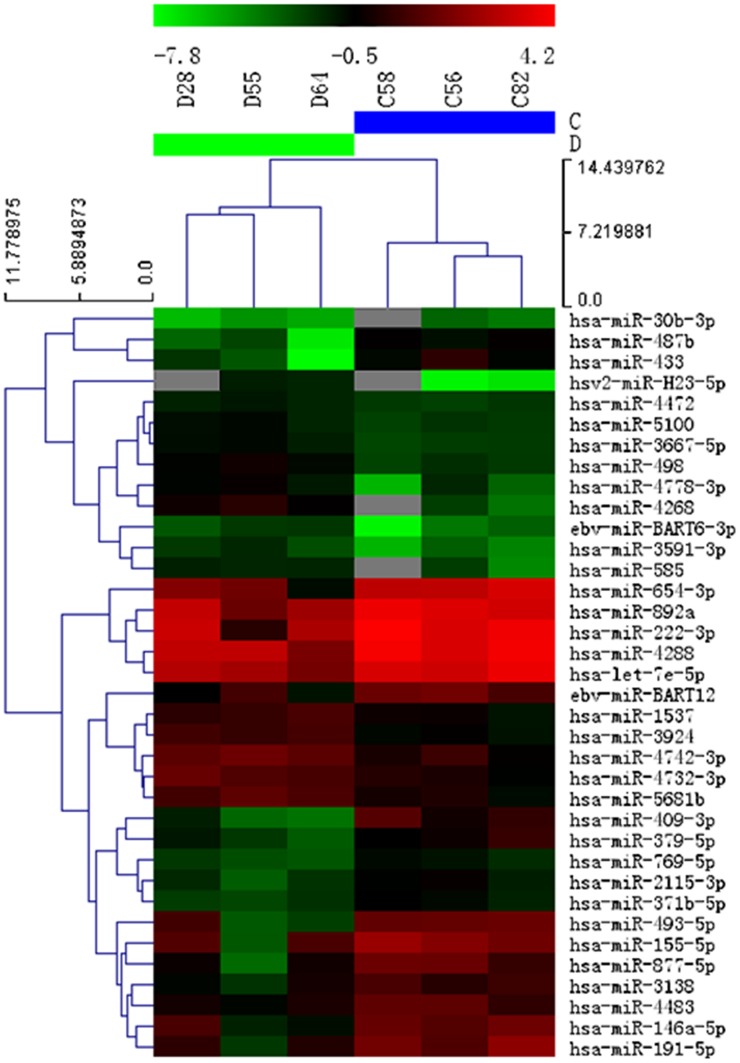
Heat map of microRNA (miRNA) microarray expression data from plasma samples of patients with ventricular septal defect (VSD) (n = 3) and VSD-free controls (n = 3). Sample miRNA species are shown at the top. The miRNA clustering tree is on the bottom. The miRNA species are shown on the right. Cluster analysis classified the samples into groups based on miRNA expression levels in each sample. The dendrogram shows significantly different expression levels of miRNAs among samples. Red indicates high expression of miRNA, and green indicates relatively low expression of miRNA.

The miRNA expression patterns in the plasma of the three patients with VSD and the three VSD-free controls were detected using miRNA microarray analysis by miRCURY LNA Array (version 11.0) to preliminarily identify miRNA expression differences between the patients with VSD and the VSD-free controls. Results showed that compared with the VSD-free controls, the patients with VSD had 36 differentially expressed miRNAs (*P*<0.05) with 15 upregulated in their expression and 21 downregulated in their expression (Table 4, Fig. 1).

**Table 4:** Differentially expressed miRNAs in patients with VSD.

Upregulated	Microarray	*P*	RT-PCR	*P*	Downregulated	Microarray	*P*	RT-PCR	*P*
miRNAs	FoldChange		FoldChange		miRNAs	FoldChange		FoldChange	
hsa-miR-4472	1.74	0.009			**hsa-miR-409-3p**	7.69	0.028	1.79	<0.001
hsa-miR-5100	2.18	0.031			hsa-miR-3138	2.44	0.029		
hsa-miR-3667**-**5p	2.56	0.010			**hsa-miR-487b**	7.14	0.003	4.17	0.025
hsa-miR-4778-3p	4.17	0.029			hsa-miR-2115-3p	2.63	0.042		
hsa-miR-3591-3p	3.90	0.039			**hsa-miR-155-5p**	2.94	0.038	2.22	0.033
hsa-miR-4732-3p	2.26	0.016			hsa-miR-30b-3p	3.03	0.033		
hsv2-miR-H23-5p	57.67	0.004			**hsa-miR-769-5p**	2.70	0.030		
hsa-miR-4742-3p	2.27	0.010			**hsa-miR-892a**	2.22	0.035		
hsa-miR-1537	2.05	0.022			hsa-miR-4483	2.38	0.038		
**hsa-miR-498**	3.05	0.006	4.33	<0.001	hsa-miR-4288	2.22	0.027		
hsa-miR-4268	6.74	0.020			**hsa-miR-379-5p**	3.70	0.040	5.56	0.013
ebv-miR-BART6-3p	3.39	0.041			**hsa-miR-222-3p**	2.63	0.042	1.96	<0.001
hsa-miR-3924	2.56	0.001			ebv-miR-BART12	2.56	0.044		
hsa-miR-5681b	2.25	0.014			**hsa-miR-654-3p**	3.70	0.010		
**hsa-miR-585**	2.70	0.045			**hsa-let-7e-5p**	2.27	0.024	3.70	0.024
					**hsa-miR-433**	6.67	0.036	10.0	<0.001
					**hsa-miR-877-5p**	3.85	0.030		
					hsa-miR-371b-5p	2.5	0.035		
					**hsa-miR-146a-5p**	2.78	0.036		
					hsa-miR-493-5p	4.00	0.018		
					**hsa-miR-191-5p**	3.57	0.043		

miRNA, microRNA; VSD, ventricular septal defect; RT-PCR reverse transcriptase polymerase chain reaction.

### Target genes of 15 in 36 miRNAs were predicted in three databases

The target genes of the 36 differentially expressed miRNAs in the VSD and control groups were predicted using three databases including targetscan, mirbase, and Miranda. Among the 36 miRNAs, 15 miRNAs corresponded with target genes across three databases. These 15 miRNAs were selected for further study (Table 4, boldfaced words).

### Expression validation by quantitative RT-PCR

**Figure 2:**
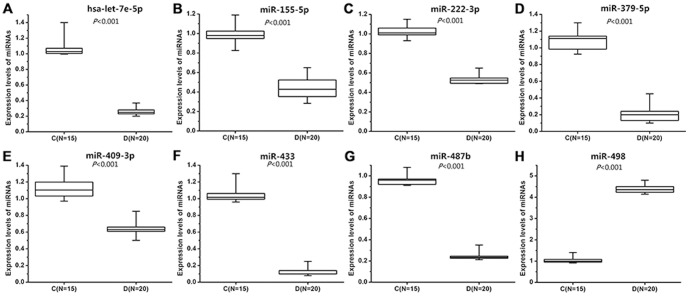
Independent validation of the differential expression of microRNA (miRNA) by reverse transcriptase polymerase chain reaction (RT-PCR) in ventricular septal defect (VSD) patients (D, N = 20) and controls (C, N = 15). hsa-let-7e-5 (*P* = 0.024),hsa-miR-155-5p (*P* = 0.033),hsa-miR-222-3p (*P*<0.001),hsa-miR-379-5p (*P* = 0.013),hsa-miR-409-3p (*P*<0.001),hsa-miR-433 (*P*<0.001),hsa-miR-487b (*P* = 0.025),hsa-miR-498 (*P*<0.001). The relative expression of eight miRNAs was normalized to expression of the internal control (has-miR-93), using the 2^−△△CT^ method.

Quantitative RT-PCR assays were performed to validate the different expression levels of these 15 miRNAs in plasma of 20 VCD patients and 15 controls. The results showed that 7 of the 15 miRNAs were not differentially expressed between the VCD patients and the controls; the remaining 8 miRNAs were differentially expressed between VCD samples and control samples, including seven downregulated miRNAs (hsa-let-7e-5p, hsa-miR-155-5p, hsa-miR-222-3p, hsa-miR-379-5p, hsa-miR-409-3p, hsa-miR-433, hsa-miR-487b) and one upregulated miRNA (hsa-miR-498)(Fig. 2). Table 5 shows representatives of target genes, mainly those involved in cardiac development (HAND1, NKX2-4, TBX-1, MAP2K4, GATA4, NOTCH1, ZFPM2, and etc.), of the 8 differentially expressed miRNAs validated by RT-PCR.

**Table 5:** Target genes associated with cardiac development predicted for 8 differentially expressed miRNAs in patients with VSD.

miRNA	Genes
hsa-let-7e-5p(downregulated)	HAND1, ABCB9, ACTA1, ANKRA2, ANKRD49, BRF2, CLP1, NKD1, NME6, TRIM71, XKR8, ZNF583, NEK3, AURKB, SLC12A9, SMUG1, ADRB2, SLC35D2, UFM1, EZH2, TIMM17B, GPR137, ZNF354A, ATP2A2, NME4, BZW2, LIPH, DTX2, BACE2, DHX57, TARBP2, GALE, MDFI, RPUSD3, CCL7, OLFM4, TRAPPC1, KIAA1539, RNF20, KLHDC8B, FAM103A1, PRTG, BZW1, GNPTAB, COIL, AP1S1, HIF3A, TTLL6, TNFSF9, PLEKHG6, GNG5, C8orf58, GATM, B3GNT1, CTHRC1, TGDS, GATA4, RTKN, USP21, LRIG2, AVEN, PRSS22, ZC3H3, KLHL6, ABCC10, YTHDF3, STXBP5, PLEKHO1, DDX19B, NID2, IL13, FASLG, PSORS1C2, NTRK3, MED28, PLD3, CDC34, MGAT4A, SPATA2, LOR, ISLR, LRIG3, ABCC5, POLL, NDST2, CDKN1A, CRCT1, CTNS, THRSP, CHRD, ZNF341, KCTD16, QARS, PQLC2, ESPL1, SLC20A1
hsa-miR-155-5p(downregulated)	ACTA1, ACTR10, BRD1, CHD7, CSF1R, DET1, FBXO11, IL13, LAMP2, MYO10, PHC2, WEE1, FOS, DHX40, HIVEP2, RNF149, TERF1, LSM14A, VPS18, SMARCA4, SALL1, BCORL1, DYNC1I1, CDC73, BAIAP2L1, H3F3A, RNF123, AICDA, TBX-1, KIAA1715, SPIN3, LRP1B, HBP1, ZBTB38, SLC12A6, USP43, STXBP5L, SAP30L, CSNK1G2, CARHSP1, TPRKB, JARID2, SGK3, PDE7A, ARRB2, C8orf4, SOCS1, USP8, KIAA1267, BOC, TRIM32, CLCN5, PTPN2, MYLK, MAP3K10, PCDH9, COL21A1, PSIP1, SDCBP, TAPT1, DNAJB7, WDR45, CEBPB, MBNL3, SKIV2L2, SPI1, DCLRE1A, FAM105A, IKBKE, SHOX, RCN2, MGP, AGTRAP, GNAS
hsa-miR-222-3p(downregulated)	ANKRD10, CDKN1B, FRAT2, MAPK10, NTF3, PCMTD1, RIT2, TOX, YTHDC1, ZFPM2, CYR61, FNDC3A, MIDN, KSR1, RSBN1L, SNCB, GRM1, MIA3, NSMCE4A, PANK3, CCDC64, ADAMTS6, GNAI2, KHDRBS2, FOS, MESDC1, RECK, VGLL4, PLCL2, KIAA1370, TBX1, HOXC10, PAIP2, LRFN2, KRT81, ZFYVE16, PCDHA4, PBX3, TP53BP2, IRX5, DMRT3, HEXIM1, MBD2, TCF7L2, RBM24, SEMA3B, ZNF181, AP3B2, SMARCA5, CDKN1C
hsa-miR-379-5p(downregulated)	CCNB1, CDKN2AIP, EIF4G2, IL28RA, KLHL14, MTM1, PCGF5, PXT1, SLC20A1, YARS, ZBTB26AKT1, GLIS1, NKX2-4, PDHX, TOP2B, UTP11L, ZDHHC20, UPK1B, GPBP1, MSH6, CWF19L1, DENND2C, SH3BGRL3, SNX13, NDN, LMO4, APOBEC2, NR4A2, EWSR1, UBE2D2, REPS1, LHX2, ATF7, PAM
hsa-miR-433(downregulated)	ABCF2, CDC27, COX6B1, GATA3, HOXA5, NOTCH1, IGFBP1, XRCC6BP1, GEMIN5, CFTR, SMG5, CLYBL, HIVEP1, RECQL, ATP5S, KIAA0195, TIPRL, EPHA5, COQ3, GPATCH8, NR2F6, SLC17A5, CENPJ, CHMP5, PPM1A, HIVEP2, TDRD5, SYNCRIP, COX8A, ATP6V0A1, NMT2, E2F3, HP1BP3, TBC1D19, ACAD8, RAD9A, FBXO5, TRIP12, PCCB, B4GALT3, SRFBP1, ADRA1A, NME5, UBR1, LRRTM2, PAK4, STMN4, WDR45L, ZC3H6
hsa-miR-487b(downregulated)	MAP2K4, NELF, ZNF219
hsa-miR-498(upregulated)	ABTB1, ANKRD11, CRLS1, JPH3, SMAD4, TEX261, TNNT1, ZEB2, KIAA1539, MAGEE1, CRH, C5, WDR47, COL1A1, MTDH, SPAG16, TIMM17A, GALNT7, PCMTD1, TRIM63, ITSN1, PAM, DCBLD2, LPP, C9orf5, BECN1, RPRM, ST8SIA2, TJP2, C10orf76, JHDM1D, SACM1L, DEDD, SLC25A12, NFIB, MGA, CACNA1A, USP34

miRNA, microRNA; VSD, ventricular septal defect.

### GO enrichment analysis for target genes

**Figure 3:**
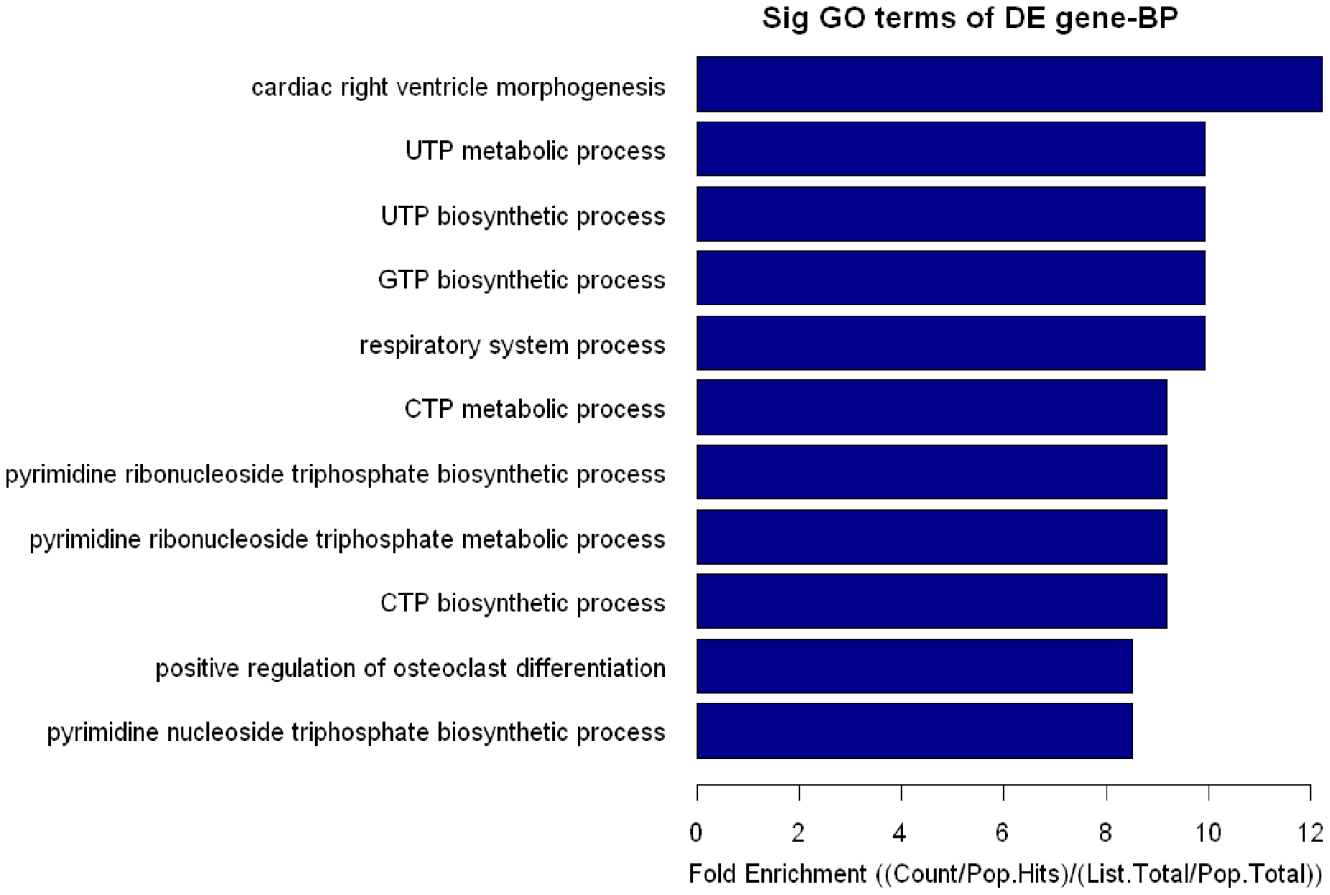
The microRNA (miRNA) Gene Ontology (GO) analysis. The blue bars represent the fold enrichment value, which is a ratio of Count/Pop.Hits and List.Total/Pop.Total. The *P*-value was calculated by Fisher’s exact test. The lower the *P*-value is, the more significant the GO Term is (*P*≤0.05 is recommended).The team of cardiac right ventricle morphogenesis’s fold enrichment value is 12.23 (*P* = 0.000235673).

The target genes predicted from patients with VSD may be involved in the pathogenesis of the VSD. GO enrichment analysis revealed that these differentially expressed miRNAs were enriched in cardiac right ventricle morphogenesis, and the top ten biological processes are shown in Table 6 and Fig. 3. The target genes NOTCH1, HAND1, ZFPM2, and GATA3 had the most enriched GO functions, and were related with cardiac right ventricle morphogenesis. They were regulated by hsa-let-7e-5p, hsa-miR-222-3p and hsa-miR-433, which were downregulated in the plasma of patients with VSD (*P*<0.05) (Table 6).

**Table 6:** Gene ontology analysis of the target genes of the miRNAs.

Term	Count	*P*-value	Genes
cardiac right ventricle morphogenesis	4	0.0002	NOTCH1, HAND1, ZFPM2, GATA3
respiratory system process	4	0.0001	ADRB2, PBX3, HOXA5, NDN
GTP biosynthetic process	3	0.0029	NME4, NME5, NME6
UTP biosynthetic process	3	0.0029	NME4, NME5, NME6
UTP metabolic process	3	0.0029	NME4, NME5, NME6
CTP biosynthetic process	3	0.0037	NME4, NME5, NME6
pyrimidine ribonucleosidetriphosphate metabolic process	3	0.0037	NME4, NME5, NME6
pyrimidine ribonucleosidetriphosphate biosynthetic process	3	0.0037	NME4, NME5, NME6
CTP metabolic process	3	0.0037	NME4, NME5, NME6
pyrimidine nucleoside triphosphatebiosynthetic process	3	0.0047	NME4, NME5, NME6

*the table only shows the top ten gene ontology (GO) terms in the functional significance list.

miRNA, microRNA; GTP, guanosine triphosphate; UTP, uridine triphosphate; CTP, cytidine triphosphate.

## Discussion

In order to evaluate the roles of miRNAs in the development of VSD this study screened differentially expressed miRNAs from patients with VSD using miRNA microarray analysis, preliminarily identified target genes regulated by these miRNAs, and provided important information for clarifying the pathogenesis of VSD at the molecular level.

In this study, we detected plasma miRNAs in three patients with VSD and three VSD-free controls by miRNA microarray analysis and found 36 differentially expressed miRNAs in patients with VSD with 21 downregulated miRNAs and 15 upregulated miRNAs (Table 4). Three miRNA target gene databases including targetscan, mirbase, and Miranda were used to predict target genes for all differentially expressed miRNAs. Among these 36 miRNAs, 15 miRNAs corresponded to 447 target genes were of general consensus across the three databases and selected for further study. Expression regulations of 8 miRNAs among these 15 miRNAs were validated by real time PCR: hsa-miR-409-3p, hsa-miR-487b, hsa-miR-155-5p, hsa-miR-379-5p, hsa-miR-222-3p, hsa-let-7e-5p and hsa-miR-433 were downregulated, while has-miR-498 was upregulated, showing the two methods gave consistent results. Among target genes of these 8 miRNAs, some were associated with cardiac development. Further functional significance analysis of these target genes showed that those associated with the most enriched GO functions regulated development of right ventricular functional morphology. Key target genes included NOTCH1, HAND1, ZFPM2 and GATA3, which were regulated by three miRNAs of the eight miRNAs validated by RT-PCR: hsa-let-7e-5p, hsa-miR-222-3p and hsa-miR-433. The expression of the three miRNAs were significantly (*P*<0.05) downregulated in the plasma of patients with VSD.

The results presented here suggested that hsa-miR-433 may regulate target genes NOTCH1 and GATA3. Previous studies have shown that NOTCH1 is closely related with ventricular development [22]. During embryonic development, NOTCH1 is expressed in the endocardium at the bottom of the trabecular layer. NOTCH1 knockout in rats causes trabecular defects [21], and NOTCH1 expression is downregulated in fetal myocardial tissue with VSD [23].

The GATA family has six genes (GATA1-6). GATA4 is a transcription factor that plays a regulatory role in a heart-mediated regulatory network, and is involved in cardiac development and expression of functional genes [24]. The GATA4 gene plays a key role in compartmentalization of the heart, atrial and ventricular development, atrioventricular valve formation, and arterial trunk separation, and it is essential for the heart to develop, mature, and play its physiological functions [25]. GATA4 may also be important for repair and reconstruction post-myocardial damage [26]. GATA3 is specifically expressed in T helper 2 (Th2) cells, and is important for Th2 cell proliferation, differentiation and development [27]. GATA3 gene expression is significantly lower in children with a combination of VSD and pulmonary arterial hypertension (PAH) than in those with PAH, so low GATA3 gene expression may be associated with VSD occurrence [28].

The hsa-miR-222-3p miRNA is involved in the regulation of the target gene ZFPM2, also known as transcription factor GATA4 (FOG2), another member of the GATA family. FOG2 is essential in heart development and can specifically interact with the GATA4 gene, inhibiting GATA4-dependent transcription [29]. FOG2 knockout mice die from heart defects on day E13.5 of the embryonic period [30], hence FOG2 may be associated with the occurrence of VSD.

Another of the four target genes predicted in our study, HAND1, is an important member of the HAND family. As a transcription factor regulating heart development, HAND2 is closely associated with ventricular muscle cell differentiation, the conduction bundle, the cardiac outflow tract, and epicardial and endometrial development [31]. HAND1 is combined with GATA4 in four binding sites of the promoter region during the regulation, both playing a regulatory role [32]. HAND1 may be involved in the occurrence of atrioventricular septal defects [33]. miR-1 HAND2 inhibition results in normal development of the heart [34]. However, little information is found on miRNA regulating HAND1 and VSD. In our study, we found that hsa-let-7e-5p was significantly downregulated in the patients with VSD. Compared with the control group, the hsa-let-7e-5p in the patients with VSD was downregulated by 0.27-fold (*P* = 0.024) (Fig. 3). This indicated that hsa-let-7e-5p may play a regulatory role in the development of VSD. The differentially expressed miRNAs might bind with specific transcription factors associated with right ventricular morphogenesis, resulting in the VSD, but this would need to be further identified by luciferase reporter gene assays.

As a new research area, miRNA regulatory mechanisms need to be explored further. It is generally considered that miRNA regulates gene expression by repressing translation or directing sequence-specific degradation of complementary mRNA. Interestingly lower expression of NOTCH1 and GATA3 is associated with VSD and yet we report reduced miRNA expression, which would in a simplistic model be expected to increase expression of these genes. It has also been suggested that miRNA may also function to induce gene expression for example, miR-373 and its premature hairpin (pre-miR-373), induced E-cadherin and CSDC2 expression by targeting specific sites in gene promoters [35]. In that case the target genes would not have been identified in this study.

While these miRNA target genes suggest that the miRNAs may be involved in the development of VSD, it is worth considering that their altered levels may also be related to secondary consequences of the VSD. This is suggested because VSDs develop early in the embryo [7], so the subjects of this study with an average age around one year will already have developed their VSD. Nevertheless, whatever the reason for their altered levels in the VSD patients these miRNAs may prove important as diagnostic markers for VSD.

Altered miRNA levels are common in a wide range of diseases and physiological processes from cancer to metabolism [8]. They have been suggested as tumor markers for gastric cancer [36], [37] and have been implicated in the development of CHD [38]–[40]. Considering the wide range of both pathological and physiological processes miRNAs are involved in [8] it is possible that the expression profiles of the miRNAs shown in this study are the result of other factors within the patient, these could be secondary results of the VSD as mentioned above or indeed other unrelated processes. To rule these factors out requires further study.

Most CHD are polygenic diseases. Many miRNA-mediated genes are involved in the development of CHD, and form regulatory networks via their interactions [41], affecting heart development. This study screened differentially expressed miRNAs in the plasma of patients with VSD, and firstly reported that hsa-let-7e-5p, hsa-miR-222-3p and hsa-miR-433 may cause cardiac abnormalities by binding with heart-specific target genes. These target genes including NOTCH1, HAND1, ZFPM2 and GATA3 may mutually interact in the development of right ventricle morphogenesis, resulting in VSD. We studied here the heart development-associated specific miRNAs by detecting the functional significance of target genes and using differentially expressed miRNA as a non-invasive diagnostic marker, and have provided a new way of thinking for further study of the etiology, early diagnosis, and treatment of VSD.

This investigation has some limitations. This study needs to be replicated in a larger, independent population to confirm the utility of these microRNAs as a diagnostic marker for VSD. The link between the differential expression of the miRNA and the target genes needs be investigated further, to provide definite evidence of their roles in development of VSD. The role of these miRNAs as potential diagnostic markers for VSD should be considered preliminary. We plan to evaluate the levels of these miRNAs in cardiac tissue from VSD repair, to ensure that they are directly related to VSD because detection in the plasma alone suggests that, potentially, they could be involved in other processes within the body.

## Conclusion

The circulating miRNA profiles of three VSD patients showed that 7 miRNAs were downregulated and 1 upregulated when matched to VSD-free controls. Analysis revealed target genes involved in cardiac development were probably regulated by these miRNAs.
